# Deterioration in glycemic control on schooldays among children and adolescents with type 1 diabetes: A continuous glucose monitoring-based study

**DOI:** 10.3389/fped.2022.1037261

**Published:** 2022-12-07

**Authors:** Yu Ding, Wenhao Zhang, Xiumei Wu, Tian Wei, Xulin Wang, Xueying Zheng, Sihui Luo

**Affiliations:** ^1^ Department of Endocrinology, The First Affiliated Hospital of USTC, Division of Life Sciences and Medicine, University of Science and Technology of China, Hefei, China; ^2^ Department of Endocrinology and Metabolic Disease, The Third Affiliated Hospital of Sun Yat-sen University, Guangzhou, China; ^3^ Institute of Endocrine and Metabolic Diseases, The First Affiliated Hospital of USTC, Division of Life Sciences and Medicine, University of Science and Technology of China, Hefei, China

**Keywords:** type 1 diabetes, children and adolescents, continuous glucose monitoring, glycemic control, schooldays

## Abstract

**Background:**

To investigate the effect of school life by comparing the glycemic control between holidays and schooldays in children and adolescents with type 1 diabetes (T1D).

**Methods:**

This observational study enrolled school-aged students with T1D (aged 6–19) from September 2019 to July 2021. Continuous glucose monitoring (CGM) records were processed and divided into holidays and schooldays. Other information was collected *via* questionnaires. We compared the results using paired T-test, Wilcoxon paired test and logistic regression analysis.

**Results:**

78 paticipants were included (40 boys, mean age 9.95 years). A total of 142,945 h of CGM data were analyzed. Overall, TIR (3.9–7.8 mmol/L) during holidays was better than schooldays [56.97 (SD 15.03) vs. 55.87 (15.06), %, *p* = 0.039]. On nocturnal (0–6 am) glycemic fluctuation, TIR was longer in children aged 6–10 [60.54 (17.40) vs. 56.98 (SD 16.32), %, *p* = 0.012] during holiday and TAR (7.8 mmol/L) was shorter [31.54 (17.54) vs. 35.54 (16.95), %, *p* = 0.013], compared with schooldays. In adolescents aged 10–19 years, TAR was also significantly shorter during holidays. Stratified analysis showed that girls, patients with longer duration, and insulin pump users had more pronounced worsening of nighttime glycemia on schooldays. Logistic regression analysis showed that girls had higher risk of worse nocturnal glycemic control [3.26, 95% CI*:* (1.17, 9.72), *p* = 0.027] and nocturnal hyperglycemia [*OR* = 2.95, 95% CI*:* (1.08, 8.56), *p* = 0.039], compared to boys.

**Conclusions:**

Children and adolescents with T1D were found to have worse glycemic control in nighttime during schooldays.

## Introduction

Type 1 diabetes (T1D) is defined as an autoimmune disease characterized by absolute insulin deficiency (1). The burden of T1D is vast and is expected to increase rapidly. In 2021, there were about 8.4 million individuals worldwide with T1D, including an estimated 1.5 million children under the age of 20 years living with T1D worldwide (2). According to the International Diabetes Registry, the hemoglobin A1c (HbA1c) of most children and adolescents with T1D does not meet the ISPAD target (3). The data from International Pediatric Registry SWEET demonstrated that only 37% of patients attained the ISPAD former HbA1c target of less than 7.5%, and 21% achieved the current goal of less than 7% (3–5).

In children and adolescents, the management of diabetes is difficult due to various factors, including physiological factors such as changes in insulin resistance related to physical growth and puberty (6). In addition, there is an increasing need for self-care knowledge and behaviors, including an understanding of carbohydrate counting, insulin calculation, self-glucose monitoring, and the effects of exercise and stress (7). A distinctive feature is that the responsibilities of children and parents in diabetes management are dynamic (7). Over time, parents have less responsibility for diabetes management. In early childhood (0–5 years), care recommendations for this age group focus on parental management (8). When children turn school age (generally 6 years of age or older), they spend most of their day at school. Children begin gaining more independence in their lifestyle, and thus diabetes management starts transitioning from a family-centered model to a patient-centered one (9). Such a shift in diabetes management responsibility is a challenge for children with T1D and affects their glycemic control (10). Together, children in lower grades in elementary school may have poor glycemic control due to poor adherence to treatment on schooldays. In contrast, children spend more time with their parents on holidays. Many studies (11, 12) show that more parental engagement is associated with better glycemic outcomes. However, glucose management can also be challenging due to the increased caloric intake on holidays (13).

However, current studies have revealed contradicting findings regarding differences in glycemic control between schooldays and holidays in children and adolescents with T1D. Several investigations of T1D indicate that children and adolescents with T1D had higher HbA1c levels (14) or lower self-monitoring blood glucose (SMBG) (15) during holidays than during school semesters, possibly because children with T1D had reduced treatment compliance (16) and a change in lifestyle. In contrast, another study demonstrated (17) no significant difference in HbA1c levels between summer vacation and school semesters. However, HbA1c levels represent a longer term of glycemic control and may be insensitive to the short-term changes between schooldays and vacation. While SMBG can make up for some HbA1c limitations, such as short-term glycemic variability, it cannot fully capture actual glycemic fluctuation (18). Continuous glucose monitoring (CGM) overcomes the problems associated with HbA1c and SMBG and offers opportunities to better reflect short-term glycemic changes and other details of glycemic variations, which assists to achieve better glycemic control.

To date, few studies have been conducted to assess glycemic control in children and adolescents with T1D, especially in regard to schooldays and holidays, using data derived from CGM. Therefore, this study aimed to investigate the impact of school life by comparing the glycemic control of CGM between holidays and schooldays among children and adolescents with T1D. This will provide important information on glycemic control in children and adolescents with T1D on schooldays.

## Methods

### Study design and participants

This was an observational study. We recruited eligible participants from the Chinese Registry of T1D, which was launched in 2014 (ChiCTR2000034642) (19). This program is conducted with the assistance of the smartphone-based application Tangtangquan (TTQ). TTQ (20) is a Chinese mobile application designed to provide diabetes self-management education and support for patients with T1D and is available for download from major application markets and the registration is free of charge. We issued advertisements in the application to recruit potential participants. Eligibility criteria were as follows: (1) patients with T1D diagnosed by an endocrinologist; (2) children and adolescents aged 6–19 years; and (3) patients who were willing to donate their CGM data for analysis in this study. The exclusion criteria included (1) refusing to participate in the study; (2) patients who wore a personal CGM device (FGM or rtCGM) for less than three days during holidays and schooldays; (3) having uncontrolled psychiatric comorbidity; and (4) currently participating in other clinical studies. The participants were divided into children (aged 6–9.9 years) and adolescents (aged 10 years or older) according to the United Nations definition of adolescence (21).

The observation period of this study was from September 2019 to July 2021. We compared the CGM-based glycemic control of the eligible participants between schooldays and holidays to investigate the impact of school life. The observation period was divided into holidays (holidays during the semester including weekends and short holidays, excluding summer and winter vacations) and schooldays. Because long vacations tended to be associated with greater lifestyle changes, such as more outdoor activities and long trips, and thus summer vacations and winter vacations were excluded. Due to the COVID-19 outbreak, Chinese students were studying from home from February 2020 to August 2020, so this period was also excluded.

### Data collection

In TTQ, a cloud platform that relies on the Nightscout system (22, 23) was established in September 2019. With this platform, TTQ users can upload their raw CGM data to the server in real time. We retrieved the CGM data of the participants during the designated study period from this platform.

We collected the following data from the participants at baseline from the T1D China Registry dataset: (1) demographic data: age, sex, education level, hometown, and household income per year; and (2) medical history: duration of T1D, age at T1D onset, diabetes complications, current HbA1c values, insulin treatment [multiple daily insulin injections (MDI), continuous subcutaneous insulin infusion (CSII), or others]. The MDI regimen consists of at least four insulin injections a day, including three premeal short-acting insulin shots and one bedtime long-acting insulin shot, and any add-on shots of short-acting insulin if necessary. In addition, information about lifestyle was obtained through a questionnaire that can be completed online or *via* telephone. Our questionnaire consists of six parts (20): (1) growth and development; (2) diet; (3) physical exercise; (4) sleep habits; (5) diabetes management; and (6) medical visits.

### Outcome measurements: CGM metrics

We use the definition of CGM metrics according to the consensus statements the ATTD Congress issued in February 2017 on 14 CGM core metrics that may be most useful in clinical practice (24). Raw CGM data obtained from the cloud platform were processed using Glyculator 2.0 software (25).

In this study, we observed the following CGM core metrics: (1) primary outcome: the proportion of time spent in the target glucose range between 3.9 and 7.8 mmol/L (TIR 3.9–7.8) and the proportion of time spent with glucose levels above 7.8 mmol/L (TAR 7.8); and (2) secondary outcome: mean glucose levels, glucose management indicator (GMI), coefficient of variation (CV), and the proportion of time spent with glucose levels below 3.9 mmol/L (TBR 3.9). The formula of GMI (%) (26) was 3.31 + 0.02392 × mean glucose in mg/dl. CV, the main metric for the evaluation of glycaemic variability, was not significantly associated with HbA1c (27). Primary and secondary outcomes were calculated utilizing data from all observed subjects from at least three days. The household income group was split based on the annual per capita income data of China (28). Duration of T1D was categorized into groups based on the average value. Additionally, we assessed dietary management compliance with midnight snacks. Daytime referred to 6 am–12 pm, and nighttime referred to 0 am–6 am.

### Statistical analysis

Data are presented as the means (standard deviations) or frequencies (proportions). Comparisons between two groups were conducted using the paired *T* test or Wilcoxon paired test, depending on whether the data followed a normal distribution. The *χ*^2^ test was used for categorical variables. Logistic regression analysis was used to analyze the association between the change in glycemic control and clinical and lifestyle-related variables. *R* (version 4.1.1) was used for statistical analyses. Statistical significance was defined as a two-sided *p* < 0.05.

## Results

### Participants and baseline characteristics

During the study period, 78 (40 boys, 38 girls) participants were enrolled. The participant characteristics are summarized in Table 1. A total of 87.2% of the participants (*n* = 68) used flash glucose monitoring (FreeStyle Libre; Abbott, North Chicago, IL, United States), and 12.8% of the patients (*n* = 10) used CGM (7 participants used Dexcom G5, 3 participants used Dexcom G6). Overall, the included patients were using CGM for 60.89% of the time during holidays, and 60.12% during school days, respectively.

**Table 1:** Baseline characteristics of participants with T1D.

Characteristics	All (*n* = 78)	Children (*n* = 43)	Adolescents (*n* = 35)	*p* value
Age (years)	9.95 (1.79)	8.57 (0.87)	11.65 (0.97)	<0.001
Gender				1.000
Boys	40 (51.28%)	22 (51.16%)	18 (51.43%)	
Girls	38 (48.72%)	21 (48.84%)	17 (48.57%)	
Onset age of T1D (years)	7.32 (2.18)	6.18 (1.35)	8.72 (2.19)	<0.001
BMI (kg/m^2^)	16.55 (2.40)	15.86 (2.30)	17.28 (2.32)	0.025
Duration of T1D (years)	2.63 (1.66)	2.39 (1.37)	2.93 (1.94)	0.175
Baseline HbA1c (%)	7.13 (1.63)	7.21 (1.89)	7.01 (1.23)	0.687
Household income per year (¥)				0.721
<100,000	19 (39.58%)	11 (44.00%)	8 (34.78%)	
≥100,000	29 (60.42%)	14 (56.00%)	15 (65.22%)	
Insulin treatment				0.567
CSII	47 (62.67%)	24 (58.54%)	23 (67.65%)	
MDI	28 (37.33%)	17 (41.46%)	11 (32.35%)	
Insulin dosage (U/kg)	0.68 (0.26)	0.68 (0.23)	0.67 (0.29)	0.903
Total CGM use time (h)	142,945	80,977	61,968	/
Holidays	48,145	26,905	21,240	
Schooldays	94,800	54,072	40,728	
CGM use time during the study period per participant [days (%)]				
Holidays	25.7 (60.89%)	26.1 (60.98%)	25.3 (59.11%)	/
Schooldays	50.6 (60.12%)	52.4 (62.23%)	48.5 (57.60%)	

Data are presented as mean (SD), or number (%). T1D, type 1 diabetes; BMI, body mass index; HbA1c, Glycated hemoglobin; CSII, continuous subcutaneous insulin infusion; MDI, multiple daily insulin injections; CGM, continuous glucose monitoring.

The participants were divided into children and adolescents, according to an age cutoff of 10 years. The average age of the patients was 9.95 (1.79) years. The average duration of T1D was 2.63 (1.66) years. The average baseline HbA1c value was 7.13 (1.63) %. More of the participants were from households with a high annual income (*n* = 29, 60.24%). Among all participants, 43 (55.13%) were in the child group (6 ≤ age < 10 years), and 35 (44.87%) were in the adolescent group (10 ≤ age ≤ 19 years). Except age, onset age of T1D and BMI, there were no statistically significant differences observed in other basic characteristics between the two groups (Table 1).

### Comparison of glycemic metrics: holidays vs. schooldays

Of the participants, a total of 142,945 h of CGM data were analyzed: 48,145 h during holidays and 94,800 h during schooldays. The characteristics of the glycemic metrics among the 78 participants are shown in Supplementary Table S1. For the whole 24-h period, TIR 3.9–7.8 during holidays was better than during schooldays [56.97 (15.03) vs. 55.87 (15.06), %, *p* = 0.039]. During the nighttime, TIR 3.9–7.8 during holidays was longer than that during schooldays [60.42 (17.06) vs. 56.92 (18.13), %, *p* = 0.001], and the TAR 7.8 during holidays was shorter than that during schooldays [31.81 (17.46) vs. 35.64 (18.55), %, *p* = 0.001]. No significant differences were found in daytime TIR 3.9–7.8 or daytime TAR 7.8 between holidays and schooldays.

**Figure 1:**
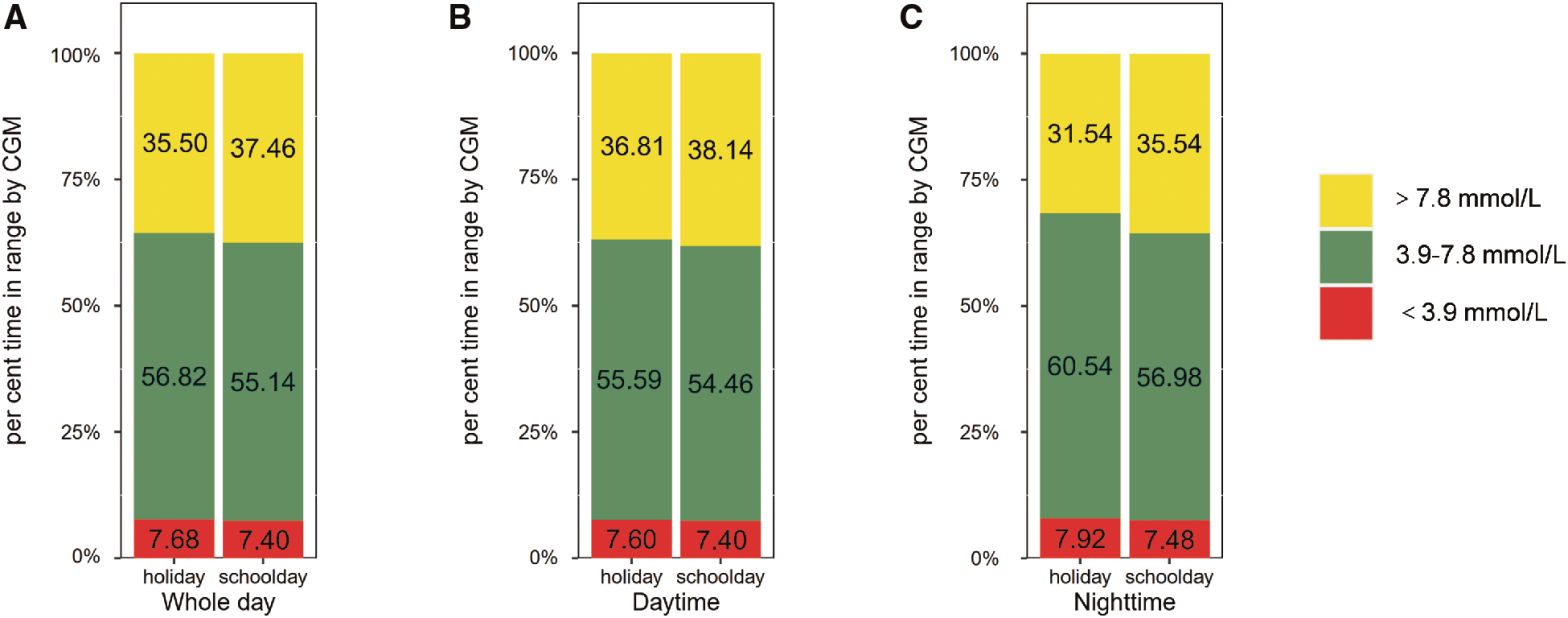
Comparison of glycemic metrics in children group: holidays vs. schooldays (**A**) Comparison of 24-hour CGM metrics between holidays and schooldays; (**B**) Comparison of daytime CGM metrics between holidays and schooldays; (**C**) Comparison of nighttime CGM metrics between holidays and schooldays; CGM, Continuous glucose monitoring; TIR 3.9–7.8, The time spent in target glucose range between 3.9–7.8 mmol/L; TAR 7.8, the proportion of time spent with glucose levels above 7.8 mmol/L; TBR 3.9, The proportion of time spent with glucose levels below 3.9 mmol/L.

**Figure 2:**
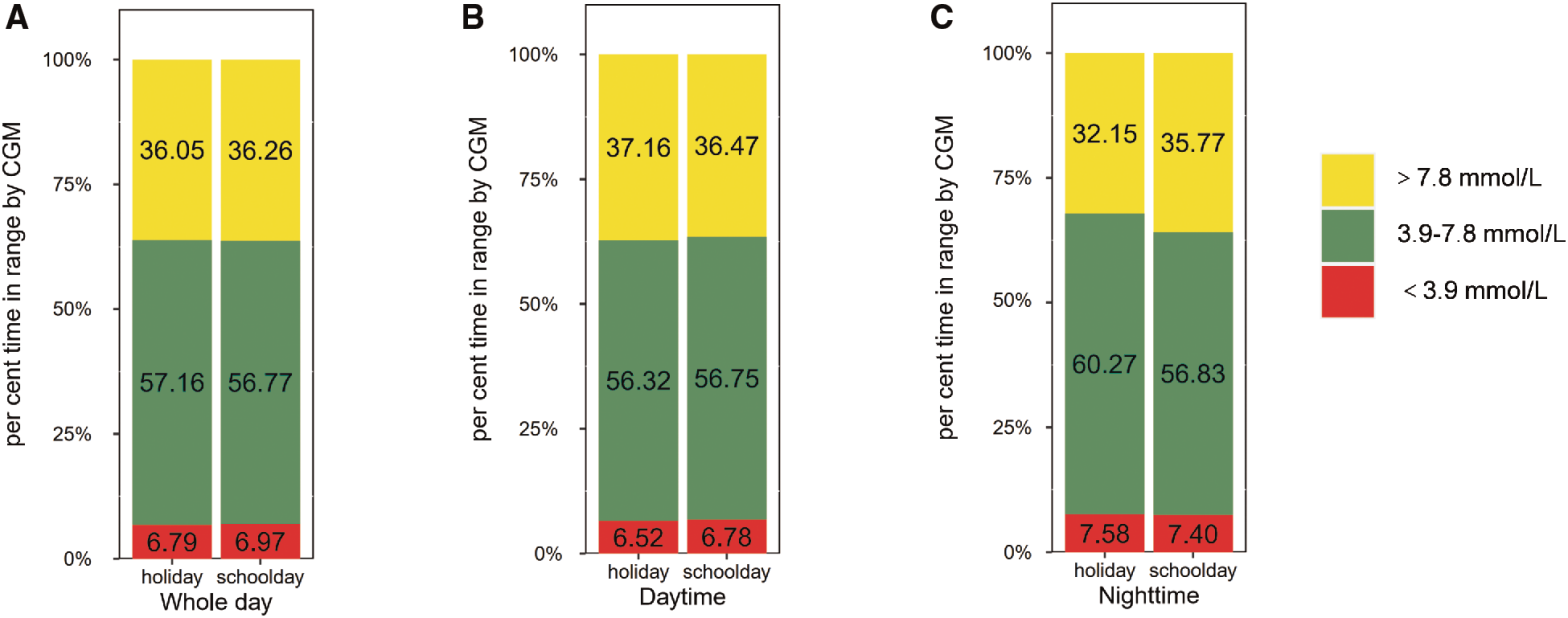
Comparison of glycemic metrics in adolescents group: holidays vs. schooldays (**A**) Comparison of 24-hour CGM metrics between holidays and schooldays; (**B**) Comparison of daytime CGM metrics between holidays and schooldays; (**C**) Comparison of nighttime CGM metrics between holidays and schooldays; CGM, Continuous glucose monitoring; TIR 3.9–7.8, The time spent in target glucose range between 3.9–7.8 mmol/L; TAR 7.8, the proportion of time spent with glucose levels above 7.8 mmol/L; TBR 3.9, The proportion of time spent with glucose levels below 3.9 mmol/L.

Then, we explored the impact of age on the difference in glycemic control between schooldays and holidays. We compared the CGM metrics between schooldays and holidays among children and adolescents. The results are presented in Figures 1,2 and Supplementary Table S2. For the child group, in the whole 24-h period, TIR 3.9–7.8 was higher during holidays than during schooldays [56.82 (14.91) vs. 55.14 (14.75), %, *p* = 0.011]. Moreover, the TAR 7.8 was lower during holidays than during schooldays [35.50 (15.20) vs. 37.46 (15.14), %, *p* = 0.017]. In the adolescent group, TIR 3.9–7.8 and TAR 7.8 also appeared to be worse on schooldays, but the differences were not significant (*p* > 0.05) for the whole day period. During the nighttime, TIR 3.9–7.8 was longer during the holidays than during schooldays in the child group [60.54 (17.40) vs. 56.98 (16.32), %, *p* = 0.012] and seemingly longer in the adolescent group [60.27 (16.88) vs. 56.83 (20.38), %, *p* = 0.051]. Additionally, the nighttime TAR 7.8 was lower during holidays than during schooldays, both in the child [31.54 (17.54) vs. 35.54 (16.95), %, *p* = 0.013] and adolescent [32.15 (17.61) vs. 35.77 (20.60), %, *p* = 0.028] groups. Notably, in the adolescent group, the mean glucose level was lower during holidays than during schooldays [7.07 (1.19) vs. 7.36 (1.47), mmol/L, *p* = 0.042], as was the GMI [6.36 (0.51) vs. 6.48 (0.63), %, *p* = 0.042]. During the daytime, there were no significant differences between holidays and schooldays for any CGM metrics in either the children or the adolescents.

### Factors associated with nocturnal glycemic stability

We further analyzed factors associated with nocturnal glycemic deterioration on schooldays, and thus, we performed subgroup analysis for nocturnal TIR 3.9–7.8, stratified by gender, duration of T1D, insulin treatment, household income, and midnight snack consumption. The results are summarized in Table 2. In the pediatric group, a difference in nocturnal TIR 3.9–7.8 between schooldays and holidays was observed among girls [children: 58.85 (16.39) vs. 64.91 (17.61), %, *p* = 0.003], with a mean difference of 6.06% in favor of holidays. However, such differences were not observed among boys or adolescents. Nocturnal TIR 3.9–7.8 was lower during schooldays than during holidays in the adolescents with a duration of T1D longer than three years [40.99 (11.06) vs. 48.46 (11.90), %, *p* = 0.033] but not among those with a duration under three years or in the child group. Children treated with insulin pumps had better nocturnal glycemic control on holidays than on schooldays [nocturnal TIR 3.9–7.8, 64.70 (18.45) vs. 61.29 (16.89), %, *p* = 0.038]. Similar results were observed in adolescents treated with an insulin pump [57.52 (14.01) vs. 52.55 (18.45), %, *p* = 0.045]. In children in the low household income group, TIR 3.9–7.8 was higher during holidays than during schooldays [65.38 (19.99) vs. 58.61 (20.90), %, *p* = 0.026]. In contrast, in adolescents in the high household income group, TIR 3.9–7.8 was lower during schooldays than during holidays [53.97 (21.73) vs. 61.62 (16.06), %, *p* = 0.017]. In the children in the no midnight snacks group, TIR 3.9–7.8 was better during holidays than during schooldays [64.26 (18.29) vs. 58.34 (19.90), %, *p* = 0.023]. In adolescents in the midnight snack group, TIR 3.9–7.8 was higher on holidays [54.98 (13.43) vs. 48.43 (15.81), %, *p* = 0.013]. The results of the subgroup analysis for nocturnal TAR 7.8 are shown in Supplementary Table S3. Similar trends were found in these findings.

**Table 2:** Associated factors with nocturnal glycemic fluctuation (TIR 3.9–7.8 mmol/L) in participants with T1D.

	Children		*p* value	Adolescents		*p* value
	Holiday	Schoolday		Holiday	Schoolday	
Gender						
Boys	56.36 (16.51)	55.21 (16.43)	0.551	64.75 (18.15)	62.14 (20.42)	0.318
Girls	64.91 (17.61)	58.85 (16.39)	0.003	55.53 (14.45)	51.21 (19.35)	0.080
Duration of T1D (years)						
<3 years	61.90 (18.64)	58.87 (17.45)	0.049	65.68 (16.20)	64.09 (19.64)	0.432
≥3 years	56.57 (13.05)	51.51 (11.40)	0.133	48.46 (11.90)	40.99 (11.06)	0.033
Insulin treatment						
CSII	64.70 (18.45)	61.29 (16.89)	0.038	57.52 (14.01)	52.55 (18.45)	0.045
MDI	57.58 (13.37)	52.84 (14.44)	0.071	66.61 (21.70)	65.20 (23.24)	0.477
Household income per year (¥)						
<100,000	65.38 (19.99)	58.61 (20.90)	0.026	57.21 (20.40)	54.17 (20.63)	0.077
≥100,000	59.36 (13.00)	56.50 (12.58)	0.126	61.62 (16.06)	53.97 (21.73)	0.017
Midnight snacks						
No	64.26 (18.29)	58.34 (19.90)	0.023	66.55 (20.89)	61.81 (25.91)	0.289
Yes	58.69 (16.26)	55.08 (13.52)	0.214	54.98 (13.43)	48.43 (15.81)	0.013

Data are presented as mean (SD). T1D, type 1 diabetes; TIR 3.9–7.8 mmol/L, Time spent in target glucose range between 3.9–7.8 mmol/L; CSII, continuous subcutaneous insulin infusion; MDI, multiple daily insulin injections.

To evaluate the associations between the aforementioned factors and the poorer nocturnal glycemic control identified during schooldays, logistic regression analysis was performed. We compared the patient's schoolday nocturnal glucose metrics with their holiday nocturnal glucose metrics. We coded the binary variable of deterioration of TIR as 1 when TIR 3.9–7.8 was lower on schooldays than on holidays and 0 otherwise. Likewise, we coded a binary variable of deterioration of TAR as 1 when TAR 7.8 was higher on schooldays than on holidays and 0 otherwise. (Details of the binary variable definition are presented in Supplementary Table S4) In our multivariate model, we adjusted for gender, age, duration of T1D and insulin treatment. The results are shown in Table 3. Girls were approximately three times more likely to have poorer nighttime glycemic control during schooldays than boys (OR = 3.26, 95% CI: 1.17–9.72, *p* = 0.027); simultaneously, girls were almost three times more likely to have a higher risk of nocturnal hyperglycemia during schooldays than boys (OR = 2.95, 95% CI: 1.08–8.56, *p* = 0.039).

**Table 3:** The relationship between the participants’ characteristics and the worse nocturnal glycemic control in schooldays by using binary logistic regression analysis.

	Deterioration of nighttime TIR		Deterioration of nighttime TAR	
Characteristics	OR (95% CI)	*p* value	OR (95% CI)	*p* value
Gender				
Boys	Ref		Ref	
Girls	3.26 (1.17–9.72)	0.027	2.95 (1.08–8.56)	0.039
Age (years)				
Children	Ref		Ref	
Adolescents	0.57 (0.20–1.57)	0.278	0.67 (0.24–1.81)	0.430
Duration of T1D				
<3 years	Ref		Ref	
≥3 years	2.60 (0.84–9.28)	0.114	2.73 (0.89–9.61)	0.093
Insulin treatment				
CSII	Ref		Ref	
MDI	0.69 (0.84–9.28)	0.488	1.00 (0.35–2.92)	0.995

T1D, type 1 diabetes; CSII, continuous subcutaneous insulin infusion; MDI, multiple daily insulin injections.

^§^Multivariate model including, gender, age, duration of T1D, insulin treatment.

## Discussion

In this observational study, we found that school-aged children and adolescents with T1D had worse glycemic control at night on schooldays. Deterioration of nocturnal glycemic control on schooldays might be affected by patients' annual household income, the type of insulin therapy, and the duration of T1D. Notably, compared to boys, girls might have a higher risk of worse nocturnal glycemic control among both children and adolescents. This finding is potentially of clinical importance, suggesting that children and adolescents should pay more attention to glycemic control during schooldays. Our results echo previous research on SMBGs (15) and show better glycemic control during holidays. Moreover, we offered more details on glycemic variations and found a lower risk of nocturnal hyperglycemia during holidays.

We observed that the children and adolescents with T1D treated with CSII had a lower TIR 3.9–7.8 during schooldays than during holidays. One possible explanation is that children and adolescents spend most of their time away from their parents, making it difficult for parents to know the details of their children's school life; hence, it is difficult to adjust the pump settings properly. For example, parents may not be able to acquire a complete list of foods consumed by their children, resulting in inaccurate carbohydrate and insulin calculations that can affect daily insulin therapy. Although recent studies (29, 30) have shown that compared with MDI, the CSII group had a lower risk of severe hypoglycemia and better glycemic control and earlier CSII use was associated with better glycemic control in younger T1D patients, our findings raise a concern about the effectiveness of CSII treatment among school-aged children during schooldays. In a previous school survey, more than one-third of adolescents hid the fact that they had diabetes at school, which could lead to reduced glucose testing and insulin omission, especially during schooldays (31). One study of children and adolescents on CSII therapy reported that 10% of participants missed mealtime boluses (32). The omission of four injections of rapid-acting insulin per week can result in a 1% rise in HbA1c. Further studies should be performed to offer more guidance for children and adolescents regarding insulin pump use at school. Similar difficulties were faced by children and adolescence in China. A report shows that the patients with type 1 diabetes in China are facing stigma, fear, and guilt may discourage insulin pump use and multiple daily injections, especially for those who require pre-meal insulin injections at school (33).

Our data showed that nocturnal glycemic control in young T1D patients during holidays and schooldays was affected by the consumption of midnight snacks. The TIR values of the children and adolescents in the no-midnight-snacks group were higher than those of the children in the midnight-snacks group, regardless of whether the snacks were consumed on a holiday or a schoolday. Midnight snacks had a more pronounced effect on adolescents' blood glucose on schooldays, and the TIR on schooldays was significantly lower than that on holidays. We also showed differences between holidays and schooldays in nocturnal glycemic control among adolescents with a longer duration of T1D, and TIR was lower on schooldays. This finding might be explained by increased independence and more time away from parents. Responsibility for diabetes management gradually shifts from parents to children and their school. The recent ISPAD clinical practice consensus guidelines state that all students with T1D, regardless of age and ability, should receive the support, encouragement and supervision of school personnel (34). In the DAWN study (35) of an international network survey with more than 6,000 people with T1D from eight countries, the results showed that respondents rated the level of support provided by schools as significantly lower than that received elsewhere. Alarmingly, a recent study found that only 2% of physical education teachers had adequate knowledge of diabetes (36). The findings of this project indicate the importance of national attention and programs for the management of diabetes in schools.

The findings showed that girls had an almost three times higher risk of worse nocturnal glucose levels and nocturnal hyperglycemia than boys. One possible explanation is that puberty typically begins at the age of 8 years in girls, which is earlier than that in boys (37). Adolescence is the transition period between childhood and adulthood, which leads to dramatic changes both physically and mentally, and girls face these challenges earlier than boys. This period is characterized by rapid sexual maturation, which usually leads to insulin resistance, exacerbating diabetic hyperglycemia (7). In addition, another possible explanation is that girls are more likely to experience reduced insulin doses during schooldays than boys (38). One study showed a gender disparity regarding the location of insulin administration at school, with girls being significantly less likely to inject insulin in the classroom and more likely to inject in the bathroom (39). This difference in injection site increased the chances of girls missing insulin doses due to classes. Therefore, we need to consider the impact of adolescence and gender when formulating treatment plans.

Previous studies (40, 41) found a link between lower annual household income and poorer diabetes knowledge. However, our study found no significant difference in nocturnal glycemic control in young T1D patients between the high and low family annual income groups. It is possible that there is a complex relationship between the family's annual income level and the patient's glucose control, and lower income is often not equivalent to worse family support.

The strength of this study is that we captured an extended time frame of CGM data reflecting glycemic control in children and adolescents with T1D during holidays and schooldays in a real-world setting. We acknowledge that there are several limitations. First, the sample size was relatively small, but the *post hoc* analysis showed that each key endpoint had more than 80% power to detect standardized effect sizes equal to 0.30. Second, on the questionnaire, some information was missing, such as growth and sexual development status. Therefore, we could not assess the associations of each of these items with nocturnal glycemic stability in the present study. Finally, as in other observational studies, even though we adjusted for several covariates related to glycemic control, residual confounding by unidentified confounders is still possible.

The results of this observational study may provide references for schools to support the self-care of diabetic patients, such as training school staff on diabetes management and strengthening psychological support for children with diabetes. They can also provide advice for clinical treatment, such as diabetes education in school-age children and individuals in early adolescence.

## Conclusions

Children and adolescents with T1D were found to have poor nighttime glycemic control during schooldays. These individuals may need more attention and guidance to improve their glucose control during schooldays.

## Data Availability

The original contributions presented in the study are included in the article/**Supplementary Material**, further inquiries can be directed to the corresponding author/s.
