# Supplementary material for: Deterioration in glycemic control on schooldays among children and adolescents with type 1 diabetes: A continuous glucose monitoring-based study
Source: Front Pediatr. 2022 Dec 7;10:1037261. doi: 10.3389/fped.2022.1037261 (PMC9768037; doi:10.3389/fped.2022.1037261)
Supplement: Supplementary file 1 [file Datasheet1.docx]

Supplementary Material

**Supplement Table 1. Overall of comparison in glycemic metrics in participants with T1D: holidays vs. Schooldays**

|  |  | Holiday | Schoolday | *P* value |
| --- | --- | --- | --- | --- |
| Whole day | TIR (3.9-7.8) (%) | 56.97 (15.03) | 55.87 (15.06) | 0.039 |
|  | TAR (7.8) (%) | 35.75 (15.48) | 36.92 (15.48) | 0.085 |
|  | TBR (3.9) (%) | 7.28 (5.90) | 7.20 (5.36) | 0.839 |
| Daytime | TIR (3.9-7.8) (%) | 55.92 (15.21) | 55.49 (14.70) | 0.462 |
|  | TAR (7.8) (%) | 36.96 (15.82) | 37.39 (15.30) | 0.566 |
|  | TBR (3.9) (%) | 7.12 (5.93) | 7.12 (5.43) | 0.996 |
| Nighttime | TIR (3.9-7.8) (%) | 60.42 (17.06) | 56.92 (18.13) | 0.001 |
|  | TAR (7.8) (%) | 31.81 (17.46) | 35.64 (18.55) | 0.001 |
|  | TBR (3.9) (%) | 7.77 (6.90) | 7.44 (6.39) | 0.579 |

Data are presented as mean (SD). Abbreviations: T1D, Type 1 diabetes; TIR 3.9-7.8, The time spent in target glucose range between 3.9-7.8 mmol/L; TAR 7.8, the proportion of time spent with glucose levels above 7.8mmol/L; TBR 3.9, The proportion of time spent with glucose levels below 3.9 mmol/L.

**Supplement Table 2. Comparison in glycemic metrics in participants with T1D: holidays vs. schooldays**

|  |  | Children | |  | Adolescents | |  |
| --- | --- | --- | --- | --- | --- | --- | --- |
|  |  | Holiday | Schoolday | *P* value | Holiday | Schoolday | *P* value |
| Whole day | TIR (3.9-7.8) (%) | 56.82 (14.91) | 55.14 (14.75) | 0.011 | 57.16 (15.40) | 56.77 (15.61) | 0.656 |
|  | TAR (7.8) (%) | 35.50 (15.20) | 37.46 (15.14) | 0.017 | 36.05 (16.04) | 36.26 (16.08) | 0.860 |
|  | TBR (3.9) (%) | 7.68 (5.82) | 7.40 (5.50) | 0.379 | 6.79 (6.05) | 6.97 (5.25) | 0.797 |
|  | Mean glucose (mmol/L) | 7.35 (1.11) | 7.43(1.09) | 0.143 | 7.37(1.11) | 7.40 (1.14) | 0.777 |
|  | GMI (%) | 6.47 (0.48) | 6.51 (0.47) | 0.143 | 6.48 (0.48) | 6.49 (0.49) | 0.777 |
|  | CV (%) | 37.47 (7.10) | 36.90 (6.35) | 0.176 | 35.67 (7.31) | 36.06 (7.59) | 0.512 |
| Daytime | TIR (3.9-7.8) (%) | 55.59 (14.98) | 54.46 (14.86) | 0.104 | 56.32 (15.70) | 56.75 (14.61) | 0.658 |
|  | TAR (7.8) (%) | 36.81 (15.53) | 38.14 (15.38) | 0.127 | 37.16 (16.39) | 36.47 (15.37) | 0.592 |
|  | TBR (3.9) (%) | 7.60 (5.94) | 7.40 (5.56) | 0.873 | 6.52 (5.94) | 6.78 (5.32) | 0.854 |
|  | Mean glucose (mmol/L) | 7.43 (1.14) | 7.49 (1.12) | 0.358 | 7.45 (1.14) | 7.41 (1.09) | 0.699 |
|  | GMI (%) | 6.51 (0.49) | 6.53 (0.48) | 0.358 | 6.52 (0.49) | 6.50 (0.47) | 0.699 |
|  | CV (%) | 37.37 (7.17) | 36.93 (6.56) | 0.323 | 35.42 (7.55) | 36.04 (7.47) | 0.397 |
| Nighttime | TIR (3.9-7.8) (%) | 60.54 (17.40) | 56.98 (16.32) | 0.012 | 60.27 (16.88) | 56.83 (20.38) | 0.051 |
|  | TAR (7.8) (%) | 31.54 (17.54) | 35.54 (16.95) | 0.013 | 32.15 (17.61) | 35.77 (20.60) | 0.028 |
|  | TBR (3.9) (%) | 7.92 (6.26) | 7.48 (6.20) | 0.352 | 7.58 (7.70) | 7.40 (6.70) | 0.878 |
|  | Mean glucose (mmol/L) | 7.10 (1.24) | 7.25(1.19) | 0.156 | 7.07 (1.19) | 7.36 (1.47) | 0.042 |
|  | GMI (%) | 6.37 (0.54) | 6.43 (0.51) | 0.156 | 6.36 (0.51) | 6.48 (0.63) | 0.042 |
|  | CV (%) | 35.42 (7.55) | 36.04 (7.47) | 0.397 | 34.45 (9.97) | 34.16 (9.56) | 0.735 |

Data are presented as mean (SD). Abbreviations: T1D, Type 1 diabetes; TIR 3.9-7.8, The time spent in target glucose range between 3.9-7.8 mmol/L; TAR 7.8, the proportion of time spent with glucose levels above 7.8mmol/L; TBR 3.9, The proportion of time spent with glucose levels below 3.9 mmol/L; HbA1c, Glycated hemoglobin; CV, Coefficient of variation.

**Supplement Table 3. Associated factors with** **nocturnal hyperglycemia (TAR 7.8mmol/l）**

|  | Children | |  | Adolescents | |  |
| --- | --- | --- | --- | --- | --- | --- |
|  | Holiday | Schoolday | *P* value | Holiday | Schoolday | *P* value |
| Gender |  |  |  |  |  |  |
| Boys | 35.99 (16.56) | 37.55 (16.70) | 0.453 | 29.49 (19.26) | 30.70 (19.35) | 0.517 |
| Girls | 26.88 (17.70) | 33.43 (17.36) | 0.009 | 34.96 (15.76) | 41.14 (21.07) | 0.027 |
| Duration of T1D (years) |  |  |  |  |  |  |
| < 3 years | 31.74 (18.86) | 34.69 (17.97) | 0.083 | 27.18 (17.27) | 29.63 (20.53) | 0.221 |
| ≥ 3 years | 30.95 (13.72) | 38.00 (14.00) | 0.087 | 42.99 (13.39) | 49.16 (13.64) | 0.042 |
| Insulin treatment |  |  |  |  |  |  |
| CSII | 28.48 (19.47) | 31.86 (17.66) | 0.075 | 36.40 (15.15) | 40.95 (19.60) | 0.037 |
| MDI | 32.80 (11.77) | 38.41 (15.08) | 0.061 | 22.28 (19.71) | 25.44 (20.38) | 0.189 |
| Household income per year (¥) |  |  |  |  |  |  |
| < 100,000 | 25.55 (21.76) | 33.25 (22.17) | 0.046 | 36.03 (21.21) | 41.18 (19.98) | 0.117 |
| ≥ 100,000 | 33.59 (11.05) | 37.00 (13.30) | 0.098 | 32.31 (16.37) | 38.53 (21.69) | 0.029 |
| Midnight snacks |  |  |  |  |  |  |
| No | 28.69 (17.77) | 34.76 (20.18) | 0.049 | 30.01 (20.44) | 35.14 (25.62) | 0.309 |
| Yes | 32.46 (17.89) | 37.50 (15.65) | 0.165 | 35.96 (17.19) | 43.41 (18.61) | 0.015 |

Data are presented as mean (SD). Abbreviations: T1D, Type 1 diabetes; CSII, Continuous subcutaneous insulin infusion; MDI, Multiple daily insulin injections; TAR 7.8, the proportion of time spent with glucose levels above 7.8mmol/L.

**Supplement Table 4.** **The definition of binary variables of deterioration of TIR and TAR**

|  | Difference value | Definition of binary variables | Meaning |
| --- | --- | --- | --- |
| TIR (schooldays) - TIR (holidays) | <0 | 1 | deterioration of TIR |
|  | ≥0 | 0 | / |
| TAR (schooldays) - TAR (holidays) | >0 | 1 | deterioration of TAR |
|  | ≤0 | 0 | / |

Abbreviations: TIR, The time spent in target glucose range between 3.9-7.8 mmol/L; TAR, the proportion of time spent with glucose levels above 7.8mmol/L;

**Holidays and Schooldays Glucose Program Questionnaire [Translated version]**

**Date of follow-up:_____________**

| **1. General information** |
| --- |
| 1.1 ID: ___________________  1.2 Gender:□ Male; □ Female  1.3 Age: ___________________  1.4 Educational status: □ Primary school; □ Middle school; □ High school; □ University and above  1.5 Place of origin: ________  1.6 Ethnicity: ___________________  1.7 Annual household income:□ 30,000 or more; □ 100,000 or more |
| **2. Growth and development history** |
| 2.1 Height: □□□□. □cm; Weight: □□□□. □kg  2.2 Height growth in 2018: ___________________cm  2.3 Height growth in 2019: ___________________cm  2.4 Presence of breast development: □ No □ Yes  2.5 Presence of testicular enlargement: □ No □ Yes  2.6 Presence of change voice: □No □ Yes  2.7 Age at first menstruation \ first ejaculation: ___________________ |
| **3. Lifestyle** |
| 3.1 Bedtime: (24hour system)  (i) Holidays: _________________; (ii) Schooldays: _________________  3.2 Wake up time: (24hour system)  (i) Holidays: _________________; (ii) Schooldays: _________________  3.3 Difficulty in falling asleep  (i) Holidays: □No; □Yes; (ii) Schooldays: □No; □Yes  3.4 Nocturnal wakefulness or early awakening  (i) Holidays: □No; □Yes; (ii) Schooldays: □No; □Yes  3.5 Toilets at night  (i) Holidays: □No; □Yes; (ii) Schooldays: □No; □Yes  3.6 Sleepiness during the day.  (i) Holidays: □No; □Yes; (ii) Schooldays: □No; □Yes  3.7 Nightmares at night  (i) Holidays: □No; □Yes; (ii) Schooldays: □No; □Yes  3.8 Duration of siesta  (i) Holidays: □ No; □ Yes: _________________h/day; (ii) Schooldays: □ No; □ Yes: _________________h/day  3.9 Duration of physical activity  (i) Holidays: □ No; □ Yes: _________________h/day; (ii) Schooldays: □ No; □ Yes: _________________h/day  3.10 Duration of study  (i) Holidays: □ No; □ Yes: _________________h/day; (ii) Schooldays: □ No; □ Yes: _________________h/day  3.11 Number of meals out  (i) Holidays: □ No; □ Yes: ____________ times/week; (ii) Schooldays: □ No; □ Yes: ___________ times/week  3.12 Number of night snacks  (i) Holidays:□No; □ Yes: _________________ times/week; _________________ (type: snacks/milk/fruit)  (ii) Schooldays: □ No; □ Yes: _________________ times/week; _________________ (type: snacks/milk/fruit)  3.13 Difficulties experienced during schooldays__________________________  3.14 Difficulties experienced during holidays_____________________________ |
| **4. Current treatment regimen** |
| 4.1 Glycated haemoglobin within the last 6 months: □ No; □ Yes (if yes, please give details)  Glycated hemoglobin level: □□. □□%  4.2 Insulin treatment: □ insulin pump (please fill in ①); □ basal + mealtime insulin (please fill in ②); □ other (please fill in ③)  ① Insulin pump  a) Name of insulin _________________  b) Total basal insulin dose □□. □□IU Pre-breakfast □□. □□IU Pre-lunch, □□. □□IU, Pre-dinner □□. □□IU  ② Basal + mealtime insulin  a) Name of basal insulin ____________, dose □□. □□IU  c) Name of mealtime insulin ____________ Pre-breakfast □□. □□IU; Pre-lunch□□. □□IU; Pre-dinner □□. □□IU  ③ Other (please elaborate) _______________________________  4.3 Oral hypoglycemic drugs: □ No □ Yes (if yes, please give details)  Name of oral hypoglycemic drug 1 ____________ (daily dose, frequency) ___________  Name of oral hypoglycemic drug 2 ____________ (daily dose, frequency) ___________ |

Completed on:□□□□year□□month□□day Completed by: ________________
